# Aminoacyl-tRNA Synthetases: On Anti-Synthetase Syndrome and Beyond

**DOI:** 10.3389/fimmu.2022.866087

**Published:** 2022-05-13

**Authors:** Angeles S. Galindo-Feria, Antonella Notarnicola, Ingrid E. Lundberg, Begum Horuluoglu

**Affiliations:** ^1^ Division of Rheumatology, Department of Medicine, Solna, Karolinska Institutet, Stockholm, Sweden; ^2^ Center for Molecular Medicine, Karolinska Institutet, and Karolinska University Hospital Solna, Stockholm, Sweden

**Keywords:** Anti-synthetase syndrome (ASSD), aminoacyl-tRNA synthetase, interstitial lung disease, myositis, autoantibodies, autoantigens, autoimmunity

## Abstract

Anti-synthetase syndrome (ASSD) is an autoimmune disease characterized by the presence of autoantibodies targeting one of several aminoacyl t-RNA synthetases (aaRSs) along with clinical features including interstitial lung disease, myositis, Raynaud’s phenomenon, arthritis, mechanic’s hands, and fever. The family of aaRSs consists of highly conserved cytoplasmic and mitochondrial enzymes, one for each amino acid, which are essential for the RNA translation machinery and protein synthesis. Along with their main functions, aaRSs are involved in the development of immune responses, regulation of transcription, and gene-specific silencing of translation. During the last decade, these proteins have been associated with cancer, neurological disorders, infectious responses, and autoimmune diseases including ASSD. To date, several aaRSs have been described to be possible autoantigens in different diseases. The most commonly described are histidyl (HisRS), threonyl (ThrRS), alanyl (AlaRS), glycyl (GlyRS), isoleucyl (IleRS), asparaginyl (AsnRS), phenylalanyl (PheRS), tyrosyl (TyrRS), lysyl (LysRS), glutaminyl (GlnRS), tryptophanyl (TrpRS), and seryl (SerRS) tRNA synthetases. Autoantibodies against the first eight autoantigens listed above have been associated with ASSD while the rest have been associated with other diseases. This review will address what is known about the function of the aaRSs with a focus on their autoantigenic properties. We will also describe the anti-aaRSs autoantibodies and their association to specific clinical manifestations, and discuss their potential contribution to the pathogenesis of ASSD.

## Introduction

Anti-synthetase syndrome (ASSD) is an autoimmune condition characterized by the presence of autoantibodies directed against an aminoacyl transfer RNA synthetase (aaRS) along with clinical features that include interstitial lung disease (ILD), myositis, Raynaud’s phenomenon, fever, mechanic’s hands, and arthritis (1, 2). ILD is the primary cause of morbidity and mortality in patients with ASSD (3–5).

Aminoacyl-tRNA synthetases (aaRSs) are a family of enzymes that catalyze the charging of amino acids onto their cognate tRNAs for protein synthesis (6). Twenty members are included in the aaRS family in most species with some exceptions (7). In humans, there are two sets of aaRSs for their actions in cytosol or mitochondria, respectively. In total, 37 aaRSs genes are encoded, which include 18 for cytoplasmic subunits (2 genes coding for separate subunits of the same aaRSs, and one gene for two fused aaRSs), 17 for mitochondrial subunits, and 2 for both sites (8). The discovery of autoantibodies against eight of these aaRS represented the first connection between aaRSs and human diseases (9).

The discovery of anti-aminoacyl-tRNA synthetase autoantibodies has allowed for the characterization of ASSD. The first detected autoantibody against an aaRS was reported in 1980 in patients with idiopathic inflammatory myopathies (IIM) (10). In 1983, Mathews et al. identified the target of Jo-1 autoantibody to be tRNA^His^ by immunoprecipitation (9). Afterwards, autoantibodies associated with similar clinical manifestations were identified against seven other aaRSs, including ThrRS, AlaRS, GlyRS, IleRS, AsnRS, PheRS, and TyrRS and were named anti-PL-7, anti-PL-12, anti-EJ, anti-OJ, anti-KS, anti-Zo, and anti-HA, respectively (11–17). At the time of discovery, it was thought that these autoantibodies identified subtypes of myositis. In 1991, Love et al. were the first that grouped patients representing distinctive clinical features with aaRSs antibodies as a unique syndrome, and in 1992, Targoff proposed to name this syndrome as ASSD (18, 19).

This review will address what is known about the function of the aaRSs and their potential autoantigenic properties. We will also describe the anti-aaRSs autoantibodies together with the associations to specific clinical manifestations and discuss their possible contribution to the pathogenesis of ASSD.

## Aminoacyl-tRNA Synthetases

**Figure 1:**
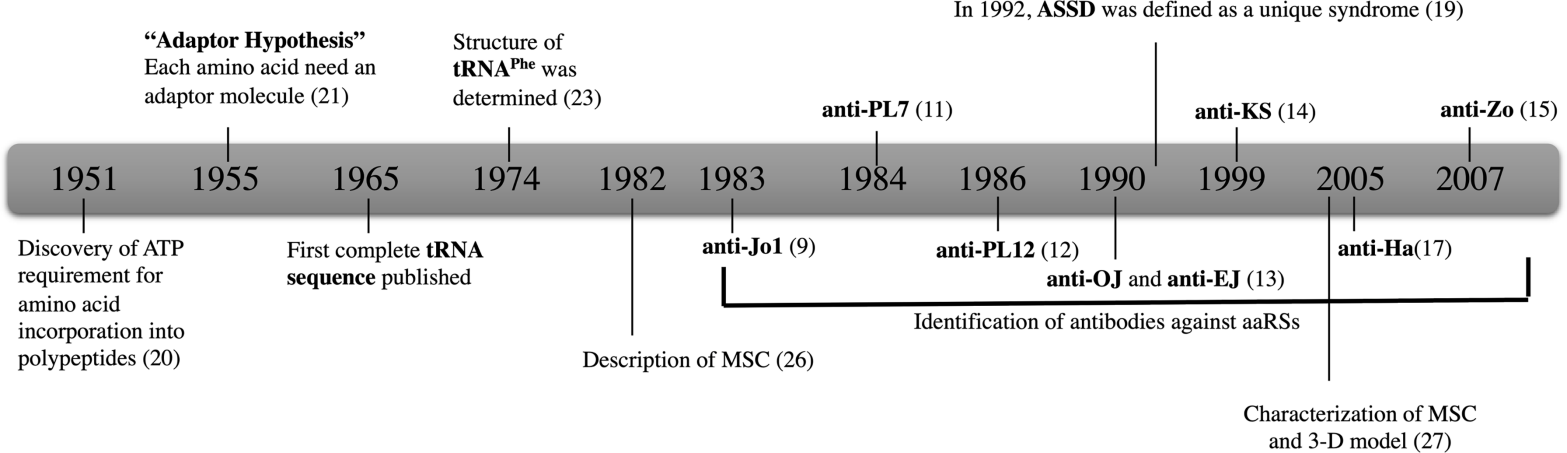
The history of aaRSs and discovery of antibodies against eight aaRSs. Anti-Jo-1; HisRS, anti-PL-7; ThrRS, anti-PL-12; AlaRS, anti-OJ; IleRS, anti-EJ; GlyRS, anti-KS; AsnRS anti-Ha; TyrRS, and anti-Zo; PheRS.

The history of aaRSs dates back to 1950s, when it was found that ATP was needed for the incorporation of amino acids to a polypeptide *in vitro* (20). Later in the mid-50s, Francis Crick introduced the adaptor hypothesis in which he proposed that each aaRS is synthesized by a unique amino acid specific enzyme (21). According to Crick, the minimum number of adaptors should be 20, one for each amino acid (22). Subsequently these adaptors were identified and are now known as tRNA molecules. The first complete tRNA sequence was published in 1965 and the structure of tRNA^Phe^ was determined in 1974 (23) (**Figure 1**).

The aaRSs are grouped into two classes: class I and class II based on distinct features of the reactions they catalyze. Class I aaRSs approach the 3’-end of their cognate tRNA with their Rossmann nucleotide binding fold-based catalytic domain (CD), whereas class II aaRSs approach their cognate tRNAs from the major groove side with anti-parallel β-sheet and flanking α-helices (24, 25). In mammals, aaRSs can also be classified as free and complex-bound forms. In fact, eight of the aaRSs (LeuRS, IleuRS, EPRS, MetRS, GluRS, ArgRS, LysRS, and AspRS) do not function as single proteins as they are part of a multi-tRNA synthetase complex (MSC) together with 3 scaffold proteins called aaRS-interacting multi-functional proteins (AIMPs) (26, 27). The MSC is thought to contribute to the cellular homeostasis maintenance in higher eukaryotes (28–30). The AIMPs also exert diverse functions other than protein synthesis, encompassing induction of synthesis of various pro-inflammatory cytokines and chemokines, angiogenesis suppression, and prevention of hyperproliferation of lung cells (29). The canonical functions of aaRSs, which include charging of tRNA synthesis, aminoacylation, and editing, are highly conserved between species. However, during the evolution from prokaryotes to vertebrates, some aaRSs acquired additional domains with characteristic structures that were not required for the canonical functions. These evolved domains, mostly found on the amino (N-) or carboxy (C-) terminus, were indicated for non-canonical activities, including translation control, transcriptional regulation, signal transduction, cell migration, angiogenesis, inflammation, and tumorigenesis (31). Evidence from recent studies suggest that either canonical or non-canonical functions of aaRSs are associated with human diseases (32).

This review will focus on the contribution of the 20 cytoplasmic aaRSs to ASSD and other diseases. To date, autoantibodies against eight of these aaRSs have been reported to be associated with ASSD. They can be listed based on the prevalence of autoantibodies against them: HisRS, ThrRS, AlaRS, GlyRS, IleRS, AsnRS, PheRS, and TyrRS (**Table 1**). Additional aaRSs that have been linked to other diseases are LysRS, GlnRS, TrpRS, and SerRS. Autoantibodies against TrpRS and SerRS have also been found in patients with other autoimmune diseases; however, the clinical features were more associated with rheumatoid arthritis (RA) or systemic lupus erythematosus (SLE) and not ASSD or myositis (33–36). We will also discuss what is known about the remaining eight aaRSs, since even though they have not yet been indicated to play a role in the pathogenesis of diseases, mutations in their encoding genes have been associated with different pathological conditions.

**Table 1:** Information on aaRSs groups based on their contribution to diseases.

Contribution to disease	Target Annotation	Class^*^	Protein name	Clinical Name	HGNC^**^ name	Non-Translational functions
**ASSD associated**	1. Histidyl-tRNA	Class II	HisRS	Jo-1	HARS	Immune Regulation, Neuronal
	2. Threonyl-tRNA	Class II	ThrRS	PL-7	TARS	Immune Regulation
	3. Alanyl-tRNA	Class II	AlaRS	PL-12	AARS	Immune Regulation, Neuronal
	4. Glycyl-tRNA	Class II	GlyRS	EJ	GARS	Immune Regulation, Neuronal, Tumorigenesis
	5. Isoleucyl-tRNA	Class II	IleRS	OJ	IARS	Immune Regulation
	6. Asparaginyl-tRNA	Class II	AsnRS	KS	NARS	Immune Regulation
	7. Phenylalanyl-tRNA	Class II	PheRS	Zo	FARS	Immune Regulation, Tumorigenesis
	8. Tyrosyl-tRNA	Class I	TyrRS	YRS/Ha	YARS	Immune Regulation, Angiogenesis, Neuronal
**Other**	9-Lysyl-tRNA	Class I–II	LysRS	KRS/SC	KARS	Immune Regulation, Neuronal, Infection, Inflammation
	10. Tryptophanyl-tRNA	Class I	TrpRS	WRS	WARS	Angiogenesis, Inflammation
	11. Seryl-tRNA	Class II	SerRS		SARS	Development
	12. Glutaminyl-tRNA	Class I	GlnRS	JS	QARS	Anti-Apoptosis
	13. Glutamyl-tRNA	Class I	LeuRS		EARS/EPRS	Inflammation
	14. Leucyl-tRNA	Class I	MetRS		LARS	Metabolism
	15. Methionyl-tRNA	Class I	ProRS		MARS	Tumorigenesis
	16. Prolyl-tRNA	Class II	ProRS		PARS	
	17. Aspartyl-tRNA	Class II	AspRS		DARS	
	18. Arginyl-tRNA	Class I	ArgRS		RARS	
	19. Cysteinyl-tRNA	Class I	CysRS		CARS	
	20. Valyl-tRNA	Class I	ValRS		VARS	

*Class I aaRSs possess a conserved amino acid sequence His-Ile-Gly-His (HIGH motif) in the amino-terminal region and a sequence Lys-Met-Ser-Lys-Ser (KMSKS or SK motif) in the carboxy-terminal region whereas Class II aaRSs do not contain specific sequence motifs (33). **HGNC, The Hugo Gene Nomenclature Committee.

## Overview of Clinical Manifestations in ASSD

According to Connors criteria, diagnosis of ASSD is made when an individual is positive for one of the eight described anti-aaRS autoantibodies (anti-Jo-1, PL-12, PL-7, EJ, OJ, KS, Ha, or Zo) and presents with at least one clinical manifestation among myositis, ILD, arthritis, Raynaud’s phenomenon, mechanic’s hands, or fever (**Table 2**) (2). The clinical spectrum of ASSD associated with the different anti-aaRSs autoantibodies is not identical but rather heterogeneous, whereby isolated or combined features are possible (46, 47). Anti-Jo-1 antibodies are found in 20%–30% of patients with IIM while those targeting other aaRS are less common, each with a prevalence below 5% (47).

**Table 2:** Common antisynthetase autoantibodies in ASSD.

Autoantibody	Protein name	tRNA synthetase	Prevalence in IIM	Clinical Manifestation	ILD*	Myositis
**Jo-1**	HisRS	Histidyl	15–30%	ILD (50–90%), fever (27–70%) Arthritis (58–75%), myositis (57%), muscle weakness (59–78%), mechanic’s hands (20–56%), Gottron’s sign (44%), RP (19–60%) (37–39).	++NSIP, OP, UIP	++
**PL-7**	ThrRS	Threonyl	5–15%	ILD (55–76%), fever (34%), myositis (48%), muscle weakness (40–52%), arthritis (31%), Gottron’s sign (41%), RP (38%) (38–40).	++UIP, NSIP, DAD	+
**PL-12**	AlaRS	Alanyl	5–10%	ILD (69–89%), fever (36–44%), pulmonary hypertension, esophageal involvement (20%), myositis (36%), muscle weakness (17%), arthritis (22–35%), Gottron’s sign (33%), RP (44%) (38–41).	++UIP, NSIP	+
**EJ**	GlyRS	Glycl	<5%	ILD (73–84%), fever (39–60%), arthritis (24%), myositis (40%), muscle weakness (39–55%), Gottron’s sign (45%), RP (13%) (38, 39, 42).	+NSIP, OP, UIP, DAD	+
**OJ**	IleRS	Isoleucyl	<5%	ILD (44–>90%), fever (13%), myositis (40–80%), muscle weakness (25%), arthritis (13–60%), mechanic’s hands (40%), Gottron’s sign (13–30%), RP (13%) (38, 39, 43).	++OP, UIP, NSIP	+/++
**KS**	AsnRS	Asparaginyl	1–8%	ILD (>90%), fever (5–8%), arthritis (26–31%), mechanic mands (30%), muscle weakness (7%), Gottron’s sign (8%), RP (31%) (14, 39, 44)	++NSIP, UIP	+
**Zo**	PheRS	Phenylalanyl	1%	ILD (77%), myositis (77%), Arthritis (66%) (44, 45) **^£^**.	+NSIP, UIP OP	++
**YRS/Ha**	TyrRS	Tyrosyl	<1%	ILD (62%), HP, Rash, arthritis (17, 45) ^€^.	+UIP, NSIP	+

*Patterns of ILD observed in ASSD **(**NSIP, Nonspecific interstitial pneumonia; UIP, usual interstitial pneumonia;OP, organizing pneumonia; DAD, diffuse alveolar damage); HP; hypersensitivity pneumonitis.^£^ Obtained from a cohort of n = 9 cases. ^€^Obtained from n = 24 cases.

ILD often dominates the clinical picture of patients with ASSD without anti-Jo-1 autoantibodies, being the initial manifestation especially in patients with anti-PL-7, PL-12, and EJ autoantibodies (48). Severity range of ILD in patients with ASSD is broad, going from asymptomatic cases to acute distress respiratory syndrome. Within ILD patterns in patients with ASSD, non-specific interstitial pneumonia (NSIP) is the most frequent, followed by organizing pneumonia (OP) and usual interstitial pneumonia (UIP). Diffuse alveolar damage (DAD) has been reported in anti-EJ patients (49). Cumulative survival seems to be higher in anti-aaRS-positive patients with ILD compared to those with idiopathic pulmonary fibrosis (IPF) (50). Within patients with ASSD, rapid progressive ILD has been observed in individuals with anti-PL-7 or anti-EJ antibodies (51). Several longitudinal cohort studies have shown that anti-PL-12 and anti-PL-7 autoantibodies are associated with more prevalent and severe ILD compared to anti-Jo-1 patients (4, 37, 38, 48, 52–54), with a lower frequency or absence of myositis (40, 55). The ILD could lead to a secondary increase in the intrathoracic pressure or lower esophageal involvement manifested by increased gastrointestinal manifestations (37, 52). The overall outcome of ILD in the group of anti-PL-7/PL-12 is worse when compared to anti-Jo-1 patients, with a higher death rate associated with lung complications (37). This lower survival rate has been associated with a delay in diagnosis, since up to 50% of the non-Jo-1 anti-synthetase patients were initially diagnosed with an overlap disorder with minimal or no evidence of myositis (38, 56). However, this mortality rate might vary as suggested by another longitudinal cohort study where, despite finding more severe lung involvement in anti-PL-7 and anti-PL-12 autoantibody-positive patients than in those with anti-Jo-1, there were no significant mortality differences between the autoantibody groups. Possible explanations for differences among the studies might be due to characteristics of the populations and disease duration before diagnosis (52).

Myositis occurs more frequently in anti-Jo-1-positive patients than non anti-Jo-1 (49). Clinically, muscle involvement may be consistent with both polymyositis and dermatomyositis, while histologically, perifascicular atrophy, a characteristic feature of dermatomyositis, although with perifascicular necrosis, seems to be characteristic for the ASSD group. In addition, electron microscopy-based nuclear actin aggregation has been seen in ASSD muscle biopsies but not in other IIM subgroups (58–60). Anti-PL-12 antibodies have also been reported in patients with immune-mediated necrotizing myopathy (61). Esophageal muscles with subsequent dysphagia are affected in one-third of patients with ASSD (62). Arthritis usually occurs at the onset of ASSD, more frequently in anti-Jo-1 compared to other ASSD autoantibody groups. The arthritis spectrum in ASSD is non-erosive rheumatoid arthritis-like with smaller joints more often involved than the larger joints, especially in case of co-occurrence of anti-aaRS and ACPA antibodies (5-14% of ASSD cases) (48, 49). Mechanic’s hands, described as erythematous and fissured hyperkeratosis of the palmar or lateral edges of the fingers, highly correlate with ASSD diagnosis, although they have been reported in other overlap myositis, especially in the presence of anti Pm-Scl and anti-MDA5 autoantibodies. Mechanic’s hands have not been reported as an isolated initial manifestation (63, 64). Other skin lesions such as Gottron’s papules/sign, heliotrope rash, shawl, holster, or V sign, typically seen in DM, have also been described in patients with ASSD (65, 66). Raynaud’s phenomenon has more frequently been observed in patients with anti-PL-12 and anti-PL-7 than in patients with other anti-aaRS autoantibodies (49, 67). Relapsing-remitting fever is one of the symptoms in 20%–60% of ASSD patients (49).

Increased risk of cancer in myositis especially in dermatomyositis has been extensively studied and reported. However, there are some discrepancies in the literature concerning prevalence of cancer in ASSD due to the varying definitions of cancer-associated myositis, the timing of malignancies, insufficient number of patients, and referral bias. Some studies show that the presence of ASSD autoantibodies, in particular anti-Jo1 and -EJ, have been associated with a lower risk of cancer (68–72), whereas some other studies and case reports describe an increased risk of cancer in anti-Jo-1-positive patients (37, 73, 74). On the other hand, a retrospective study including a small cohort showed that being male, over the age of 60, and the coexistence of anti-SSA/Ro autoantibodies along with ASSD were risk factors for the development of neoplasm (51). Hence, larger studies with proper age-matched controls are required to determine if there is an association between cancer and ASSD. Moreover, systematic reviews and meta-analysis recommend careful cancer screening in PM/DM patients with ILD, especially those with multiple risk factors for malignancy (75).

## Autoantigenic aaRSs in Anti-Synthetase Syndrome and Clinical Associations

There are eight yet identified aaRSs that have become targets of the immune system with the development of autoantibodies and the clinical ASSD. The pathogenic mechanisms for this syndrome are not clear. The individual aaRSs have somewhat different properties and functions within the cells and extracellular functions. Here, we give an overview of these eight aaRSs and detailed clinical associations to the corresponding autoantibodies (**Table 3**).

**Table 3:** tRNA synthetases, epitopes and immune activities.

Autoantigen	Immune modulatorydomains of the protein	Immune activities
1.HisRS	GrB B cleavage site: **LGPD^48^-E** **Immunogenic peptide**: **VKLQGERVRGLKQ** **Immunogenic region:** N-terminal 151 amino acids **Immunogenic site: **N-terminal domain, WHEP Domain	T-cell proliferation (76). N-terminal domain: chemoattracts lymphocytes and immature dendritic cells trough interaction with CCR5 (77, 78). **VKLQGERVRGLKQ** peptide: CD40L upregulation in CD4^+^T cells, with cytokine production of IFNg, IL-2, IL-17 (78).
2.ThrRS	Secreted ThRS has autocrine and possibly paracrine functions	Stimulate endothelial cell migration and angiogenesis. Activation and maturation of DC, Upregulation of CD4^+^ and CD8^+^ T cells, and increased IFN-g secretion (79, 80).
3.AlaRS	Nine-base region of the anticodon loop **Immunoreactive region:** amino acids 730-951	Astrocyte IL-6 release, hMSC differentiation (81) Human AlaRS shows substantial mischarging activity, which can generate mistranslated proteins that can potentially participate in cellular stress responses and adaptations (82–84).
4.GlyRS	N-terminal domain	Secretion from macrophages in response to Fas-L from tumour cells (85, 86).
5.IleRS	GrB B cleavage site:**VTPD^983^-Q** Quaternary interactions in the MSC	Promote cell migration /o cytokine release (57, 87).
6.AsnRS	GrB B cleavage site: **VAPD^632^-R**	Activation via CCR3+ CCR5+ chemokine receptors (87).
7.PheRS	α subunit	Stimulates cell proliferation,cell differentiation (88).
8.TyrRS	N- and C-terminal domain Truncated mini-TyRS: **Met^1^-AsP^343^** PMN elastase cleavage site: **Pro^344^-Ser^528^**	**C-terminal domain:** migration of monocytes and stimulation of TNFa. Includes the amino acid sequence **Pro^344^-Ser528** associated with MP and PMN chemotaxis. Truncated mini-TyRS **Met^1^-AsP^343:^** chemoattractant only for PMN. **N-terminal:** migration of PMNs in a dose dependent manner. Possible functional correlation with IL-8. Can be present in platelets, playin a role in monocyte/macrophage differentiation during bacterial infection (42).
9.TrpRS	N-terminal domain is cleaved under the catalysis of Mmp7/Mmp8, generating the peptide **^1-47^WRS**, unable of activating TLR2/TLR4	IFN-g induces the secretion of TrpRS by macrophages, endothelial cells and fibroblasts. High expression in CD4T cells can resist IDO-mediated immunosuppresion from DC in Grave's disease (89). Secretion by monocytes upon infection. Interacts with TLR2/TLR4 leading to the secretion of TNFa and IL-8, neutrophil infiltration and phagocytic abilities (90–92).
10. LysRS	N- and C-terminal domain	Presence of phosphorylated KRS in activated mast cells (93). Caspase-8 mediated the release of LysRS from tumor cells and the released KRS induced macrophage migration. Secretion via exosomes or exosomes-like extracellular vesicles (94).

GrB, granzyme B; DC, dendritic cell, MSC, multienzyme synthetase complex; hMSC, human mesenchymal stem cells; IDO: indoleamine 2,3-dioxygensase; PMN: polymorphonuclear cells; MN, mononuclear phagocytes; Mmps, matrix metalloproteinases.

### Histidyl-tRNA Synthetase

**Figure 2:**
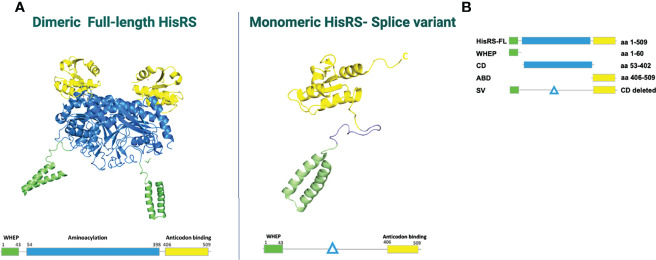
**(A)** HisRS structure visualized using PDB ID: 4G84 and 2LW7 (98). **(B)** Schematic figure of HisRS constructs, adapted from Notarnicola et al. (99). HisRS-FL, HisRS-full length; WHEP, WHEP domain; CD, catalytic domain; ABD, anti-codon binding domain; SV, splice variant.

Histidyl tRNA synthetase (HisRS) is a homodimeric enzyme, whose location is mainly cytoplasmic. HisRS is responsible for the incorporation of histidine into a growing peptide (95–97). The N-terminal fragment or CD includes the residues 1–320 and is responsible for the specific aminoacylation of tRNA (98). The C-terminal fragment is essential for the dimeric structure of the enzyme. Additionally, at least two HisRS-splice variants (SV) have been identified lacking the CD (**Figure 2**). Besides the intracellular cytoplasmic location of HisRS, this enzyme can also be found in the extracellular compartment (98), although its extracellular functions have not been fully clarified. Both *in vitro* and animal studies have suggested that HisRS is involved in several regulatory mechanisms of cell metabolism and the regulation of immune responses. Howard et al. have demonstrated that the N-terminal domain serves as a chemoattractant for naïve lymphocytes and immature dendritic cells (DCs) through interaction with CCR5 (77). The N-terminal has also been considered to be the immunodominant epitope, specifically the 1–60 amino acid fragment (100). In particular, *in vitro* studies performed in myositis samples showed that T-cell stimulation assays using a 13-mer peptide from the HisRS N-terminal domain elicited an inflammatory response in blood and bronchoalveolar lavage fluid (BALF) T cells (78). Interestingly, *in vivo* the levels of extracellular HisRS were higher in sera of anti-Jo-1-negative patients compared to healthy individuals and were almost undetectable in patients with Jo-1 antibodies. This observation suggested that another possible immune mechanism could be involved, such as the formation of immune complexes of autoantibodies with the protein, but this still needs to be confirmed.

Anti-HisRS autoantibodies (anti-Jo-1) were initially identified in 1983 by Mathews M. and Bernstein R (9). Anti-Jo-1 autoantibodies are the most common among the myositis-specific autoantibodies (MSAs) with frequencies between 20% and 30% in patients with IIM (101–103).

Worldwide epidemiological studies have shown that patients presenting with anti-Jo-1 autoantibodies can develop ILD in up to 90% of the cases (38, 41, 104). In the American and European Network of Antisynthetase Syndrome (AENEAS) cohort study of anti-Jo-1-positive patients, ILD was present in 50% at disease onset and in 84% after 80-month follow-up (67).

### Threonyl-tRNA Synthetase

Threonyl-tRNA synthetase, ThrRS, also referred to as TRS and the target of anti-PL-7 autoantibodies, is an aminoacyl-tRNA synthetase that catalyzes the aminoacylation of tRNA by transferring threonine. Besides its essential function of catalyzing aminoacylation, ThrRS can be secreted into the extracellular compartment where it can have other non-canonical functions (105). In particular, the extracellular secretion of ThrRS has been associated with the exposure of vascular endothelial cells with proinflammatory cytokines such as tumor necrosis factor-a (TNF-a) or vascular endothelial growth factor (VEGF). Additionally, *in vitro* and *in vivo* assays have shown that ThrRS stimulates endothelial cell migration and angiogenesis, indicating that ThrRS can acts as an angiogenic pro-migratory extracellular signalling molecule (79).

A recent study showed that ThrRS can induce maturation and activation of DCs with a Th1 response *in vitro* and *in vivo*, upregulation of CD4^+^ and CD8^+^ T cells, increased IFN-gamma secretion associated with viral clearance in influenza virus (H1N1)-infected mice, and increased IL-12 production by the MAPK/NF-kB pathways. Interestingly, CD4^+^IFNg^+^ and CD8^+^IFNg^+^ T cells and IFNg levels of ThrRS-DC immunized mice were significantly upregulated in BALF compared with the control group (80).

In the muscle, ThrRS may play a role as negative regulator in myogenic differentiation, by inhibiting Axin1, through the kinase c-Jun N-terminal (JNK), a downstream target of ThrRS. In particular, the presence of UNE-T or TGS domains was necessary for ThrRS myogenic function (106). Further studies analyzing the role of this protein in the innate and immune response as well as the ThrRS expression in muscular diseases are still needed to further understand the role of this aaRS in the disease.

There are few reports on clinical presentations of patients specifically with anti-PL-7 autoantibodies. In a European multicenter study, the frequency of anti-PL7 was 1.87% (18/964) (107), whereas a single-center retrospective cohort from China reported that anti-PL-7 autoantibodies had the same frequency (27% 30/108) as anti-Jo-1 autoantibodies (30.5% 33/108) (108). In Asian studies, the most common ILD pattern observed in anti-PL-7-positive patients was NSIP (15%), which was in line with an earlier study that reported mainly NSIP patterns (50%) followed by OP (41.7%) (109). In this study, UIP was only observed in the anti-PL7-positive group (4%) and was associated with a low response to therapy (108). In addition, the presence of this autoantibody predicted the long-term deterioration in ILD (110).

### Alanyl-tRNA Synthetase

In contrast to most aaRS, alanyl-tRNA synthetase (AlaRS), the target of anti-PL-12 autoantibodies, recognizes its substrate in an anticodon-dependent manner with recognition of a single G3:U70 wobble base pair, which is the dominant identity determinant for tRNA aminoacylation (82). This simplified mechanism increases the likelihood of mischarging by AlaRS and has been associated with neurodegenerative disorders such as Charcot-Marie-Tooth disease (CMT) type 2, which is characterized by axonal peripheral neuropathy with muscle weakness, wasting, and impaired sensation in the extremities (82).

Autoantibodies against alanyl-tRNA synthetase, also known as anti-PL-12, was the third myositis-specific autoantibody identified in 1986 by Bunn et al. (12). Unlike anti-Jo-1 and anti-PL-7 autoantibodies, anti-PL-12 IgG recognizes independently two antigens: alanyl-tRNA synthetase and its cognate tRNA^Ala^, suggesting that this recognition occurs by separate autoantibodies. The antigenic epitope is located in the anticodon region (83), and an epitope mapping of this protein identified the immunoreactive region outside the CD, within amino acids 730–951 (111) (**Table 2**). In the case of anti-PL-12 autoantibodies, the severity of the clinical manifestations is mainly driven by the ILD, which was notable in cases associated with pulmonary hypertension (55). Both in American and European reports, a series of anti-PL-12-positive patients showed a low prevalence of muscular involvement, mechanic’s hands, and Raynaud’s phenomena (55, 112, 113). Regarding the histopathologic and radiographic features, UIP is the most common pattern of lung involvement (114).

### Glycl-tRNA Synthetase

Glycyl-tRNA synthetase (GlyRS), the target of anti-EJ autoantibodies, is a dual-localized homodimeric aaRS, found in both the cytoplasm and the mitochondria. It catalyzes the attachment of glycine to its cognate tRNA. Mutations in the gene coding for human GlyRS are associated to neurodegenerative diseases including the distal spinal muscle atrophy type V and CMT disease (115). The impairment in the mitochondrial metabolism in neurons is one of the mechanisms through which mutations in GlyRS lead to neurological diseases (116). GlyRS has been also shown to circulate in serum of human subjects but, in contrast to HisRS, extracellular levels of GlyRS were not found to be significantly different between healthy individuals and patients with myositis (97). *In vitro* experiments have demonstrated that it can be secreted from macrophages in response to Fas ligand that is released from tumor cells (85). Therefore, it has been suggested that GlyRS is involved in immune surveillance against cancer (85).

Autoantibodies against GlyRS (anti-EJ) were described for the first time in 1990 by Targoff in patients with myositis and ILD (13). In anti-EJ-positive patients from the AENEAS cohort and a large Chinese cohort, ILD was the most frequent clinical manifestation, being isolated in almost 40% of the patient group (48, 117). ILD has been reported as an early manifestation of the anti-EJ-ASSD disease course (117, 118). Regarding ILD patterns, NSIP, UIP, OP, and DAD have been described in patients with anti-EJ antibodies (117–119). OP was also found to be an independent risk factor for developing rapidly progressive ILD (117). Among other ASSD features, fever, mechanic’s hands, and Raynaud´s phenomenon have been reported as accompanying findings (48, 117, 119).

### Isoleucyl-tRNA Synthetase

Isoleucyl-tRNA synthetase, IleRS (IARS), the target of anti-OJ autoantibodies, is a component of the multi-enzyme complex (MSC) described above that is important for the stabilization of the interactions between the components (28–30, 120). In addition, IleRS is important for protein synthesis and signaling pathways.

The anti-isoleucyl-tRNA synthetase autoantibody (also known as anti-OJ) was identified in 1990 and was initially described in a patient with a history of severe, progressive pulmonary fibrosis and pulmonary hypertension (13). This autoantibody has a low prevalence (<5%) among patients with IIM (57). Anti-OJ autoantibodies react with lysyl-tRNA synthetase (KARS) and the epitope of anti-OJ autoantibodies could be based on quaternary interactions between MSC components (57), making the detection in the clinical practice problematic. The low frequency of this autoantibody might be associated to the elusiveness of the primary target(s) of anti-OJ. In the clinical setting, multiplex assays based on immunoblotting such as line immunoassay (LIA) and dot blot assays (DBA) have poor performance of anti-OJ (121, 122). Anti-OJ autoantibodies have also been difficult to detect in ELISA, even with the use of recombinant proteins from *Escherichia coli* and Hi-5 (insect cell line), suggesting that either anti-OJ autoantibodies might recognize other components of the MSC or that the structural conformation of the complex is important for the recognition by this autoantibody (123). To date, immunoprecipitation (IP) remains the preferred method for anti-OJ autoantibody detection.

A review of 52 published cases identified ILD as the main clinical manifestation in 90% of the anti-OJ positive cases, with the patterns of UIP, OP, and NSIP being the most frequent. Prevalence of myositis seems to vary according to the criteria applied for ASSD classification and the ethnicity of the population included in the studies. In an Asian cohort, the frequency of severe myositis was reported to be as high as 57% (62), while in the AENEAS cohort study, 40% of the patients with anti-OJ autoantibodies had hypomyopathic forms of ASSD or never developed myositis (124). Arthritis, fever, Raynaud’s phenomenon, and mechanic’s hands are also present, but in a lower frequency, being an incomplete presentation of the ASSD frequent (57).

### Asparaginyl-tRNA Synthetase

The asparaginyl-tRNA synthetase (AsnRS), the target of anti-KS autoantibodies, catalyzes the attachment of asparagine to its cognate tRNA during translation. As for non-canonical functions, AsnRS has been shown to be involved in growth regulation mediated by the Hippo signaling pathway (a pathway involved in growth regulation, dysregulation observed in many cancers) and, therefore, possibly implicated in tumorigenesis (125). Studies also reported pro-inflammatory functions of AsnRS. In particular, AsnRS was shown to induce CCR3-expressing cells to migrate and, like HisRS, chemoattract DCs (77). The non-translational chemokine activity of AsnRS is thought to be exerted by an additional domain at the N-terminal of the protein sequence, not present in the prokaryotic system. The modulating activity of AsnRS on CCR3 signaling has been suggested to be implicated in the pathophysiology of ASSD and ILD (126). Similar to GlyRS, AsnRS has been detected in extracellular compartments and serum levels were not significantly different between myositis patients and healthy individuals (97).

Autoantibodies against AsnRS (anti-KS) occur in less than 5% of patients with IIM and were first described in 1999 in two patients with ILD and no evidence of myositis (14). A review of the published literature about the clinical features associated with anti-KS autoantibodies has shown that ILD with NSIP or UIP patterns is the dominant feature, being the only manifestation in 50% of patients (63). Myositis seems to rarely affect this group of patients, while arthritis, Raynaud’s phenomenon, and mechanic’s hands may occur in one quarter of them (127)

### Phenylalanyl-tRNA Synthetase

Phenylalanyl-tRNA synthetase, PheRS, the target of anti-Zo autoantibodies, has been linked to cancer by reports of increased expression in some cancers (128–130), and suggested to be a prognostic indicator for some cancers (131). Thus, expression of PheRS was increased in gastric cancer tissue and the levels of expression correlated with distant metastasis and poor survival (132). Mechanistically, PheRS regulates anti-apoptotic signaling and cell proliferation through its upstream interaction proteins (133); however, the regulation of oncogenesis and development of gastric cancer by PheRS need further investigation.

The presence of autoantibodies against PheRS, anti-Zo, was first described in 2007 in a patient with ASSD (15). Anti-Zo is a rare anti-synthetase autoantibody. The largest cohort positive for anti-Zo autoantibodies was a case series of nine patients in UK (134). Seven (78%) of the patients had ILD, and two patients had evidence of muscle involvement, suggesting that anti-Zo autoantibodies are associated with features of ASSD. Moreover, two-thirds of the patients had autoantibodies against anti-Ro52, which has been previously reported to co-exist with other anti-synthetase autoantibodies and more severe ILD (135, 136). A more recent study reported the prevalence of anti-Zo autoantibodies as 1.4% in patients with ILD and novel associations of anti-Zo with connective tissue-disease related ILD (CTD-ILD) and idiopathic pneumonias (45).

### Tyrosyl-tRNA Synthetase

Tyrosyl-tRNA synthetase or TyrRS, the target of anti-YRS/anti-HA autoantibodies, can split into two separate fragments, and these fragments have distinct cytokine activities whereas the full-length TyrRS is inactive for cytokine activities (42, 137). Secretion of both fragments are induced by leukocyte digestion and active forms of TyrRS can be naturally generated by alternative splicing or proteolytic cleavage (42, 137). The fragment that includes the C-terminal domain induced migration of mononuclear phagocytes and stimulated production of TNFa. On the other hand, the N-terminal domain induced migration of polymorphonuclear leukocytes (PMNs) in a dose-dependent manner very similar to the CXC chemokine interleukin -8 (IL-8) (42). Similarities in the effects of IL-8 and mini TyRS (N-terminal) on PMNs indicate a possible functional correlation between mini TyrRS and IL-8 activity. In addition, human mini TyrRS induced angiogenesis *in vivo* in different organisms similar to IL-8 (138). The N-terminal domain of TyrRS was also reported to be present in platelets, from which they are released by unknown mechanisms and to regulate monocytes/macrophage differentiation during bacterial infections (139).

The first report of autoantibodies against TyrRS referred to as anti-YRS or anti-HA was published in 2005 in a patient with ASSD features (17). The prevalence of anti-HA autoantibodies in a large cohort of patients with ILD (*n* = 1,194) was 2% (45).

## Possible Autoantigens in Other Diseases

### Lysyl-tRNA Synthetase

As mentioned previously, aaRSs gained additional domains and functions throughout evolution. One example is Lysyl-tRNA synthetase (LysRS), also referred to as KRS or SC. LysRS binds to macrophages and monocytes leading to macrophage migration and TNF production when present in the extracellular media (140). Moreover, there are many reports in the literature indicating a role for LysRS in human immunodeficiency virus (HIV) replication, signal transduction, and neurodegenerative diseases (141). The mechanism of how LysRS contributes to HIV replication has been well studied, and it has been established that HIV recruits LysRS to serve as the reverse transcriptase through the interaction of LysRS and Gag protein (142–149). In addition, the contribution of LysRS in cancer has been established by many studies. LysRS has been shown to promote cancer metastasis by inducing cancer cell migration through the interaction with 67LR protein (150). LysRS can be secreted by cancer cells to induce inflammatory responses (151), whereas a more recent study showed a novel mechanism for the secretion of LysRS *via* exosomes or exosome-like extracellular vesicles from cancer cells, which is controlled by a caspase-8-dependent pathway (94).

There is also a possible connection of LysRS with amyotrophic lateral sclerosis (ALS). In some patients with ALS, a mutation in SOD1 is observed. The mutation in SOD1 induces apoptosis of motor neurons, thus leading to the onset of neurodegeneration and interestingly LysRS associates with mutant but not wild-type SOD1 (152). The abnormal interaction between SOD1 and LysRS contributes to mitochondrial dysfunction in ALS (153). In addition, a loss-of-function mutation in the CD of LysRS was implicated in CMT disease. It was reported that the mutation in LysRS severely affects the enzyme activity (154).

### Glutaminyl-tRNA Synthetase

Glutaminyl-tRNA synthetase (GlnRS) is one of the two enzymes in this family that is not found in all organisms, such as Gram-positive eubacteria, archaebacteria, and organelles, suggesting that this aaRS has evolved along independent evolutionary pathways (155). Additionally, GlnRS appears to be the largest polypeptide in the human multienzyme synthetase complex and shares three significant regions of sequence similarity with the translation elongation factor EF-1 (156).

In humans, it is bifunctional, being specific for two amino acids (glutamine and proline) (28), acquiring the term glutamyl-prolyl-tRNA synthetase [Glu-ProRS or EPRS (157)]. Among the non-canonical functions of GlnRS is the ability to block apoptosis trough a negative regulation of the apoptosis signal-regulating kinase 1 (ASK1) (158). Additionally, *in vivo* studies in a rat model to evaluate changes in sensory neurons after nerve injury showed that the expression of GlnRS was decreased in the dorsal root ganglia (DRG). Thus, this aaRS may play a potential role as a neurotransmitter; however, further research is required (159). In addition, GlnRS expression may affect macrophage recruitment to injured DRG neurons (159–161).

Mutations in the gene encoding GlnRS have been associated to early-onset epileptic encephalopathy (162, 163). GlnRS deficiency has been associated with neurodegenerative disorders associated with severe developmental delay, microcephaly, delayed myelinization, and intractable epilepsy, which seems to be more severe than other disorders associated with mutations in tRNA synthetases (164).

### Tryptophanyl-tRNA Synthetase

Mammalian tryptophanyl t-RNA synthetase, TrpRS or WRS, has gained functions such as alternative splicing and proteolytic cleavage through evolution. This aaRSs is found not only in the cytosol but also in the nucleus. In the cytosol, it plays a role in innate immune responses, angiogenesis, and type II IFN signaling.

TrpRS is secreted into the extracellular milieu by monocytes upon infection with certain pathogens and it interacts with TLR2 and TLR4 leading to the secretion of TNFα, neutrophil infiltration, and increased phagocytotic abilities (90–92). These responses help to clear out infections at the early phase, indicating the importance of TrpRS as a ligand for immune regulation through TLR signaling. In support of this hypothesis, high levels of TrpRS were found in sera of patients with sepsis, a potentially fatal complication due to infection, when compared to healthy controls (92). Additionally, increased TrpRS expression from CD4^+^ T cells resisted indoleamine 2,3-dioxygensase (IDO)-mediated immunosuppression from DC in Graves’ disease (89). Autoantibodies against TrpRS have been found in patients with autoimmune diseases, where the clinical features were associated with rheumatoid arthritis (33–35).

### Seryl-tRNA Synthetase

There are few studies investigating the non-canonical functions of seryl-tRNA synthetase, SerRS. It was reported that SerRS interacts with a transcription factor called Yin Yang 1, to form a complex that negatively regulates vascular endothelial cell growth factor A during angiogenesis (165)

Autoantibodies against SerRS have been detected in a few cases of systemic lupus erythematosus or rheumatoid arthritis but not in myositis (36, 77, 166).

## Others

So far, there are no reports in the literature about the remaining eight aaRSs being recognized as autoantigens. However, recently, several mutations in encoding genes in mitochondrial or cytosolic compartments have been identified. These mutations resulted in dysfunctional aaRSs that lead to a variety of multi-organ, neuronal, and metabolic disorders (**Table 4**) (8). Fifty-five percent of mutations in genes encoding for mitochondrial aaRSs were associated with disease, whereas in cytoplasmic aaRSs, the percentage is 20% (177). Notably, mutations in genes encoding for mitochondrial aaRS are often associated with some form of myopathy (**Table 4**).

**Table 4:** Mutations in genes encoding mitochondrial or cytosolic aaRSs and associated disorders (8).

Aminoacyl tRNA-synthetase	HGNC name	Mitochondrial	Cytosolic
1. AspRS	DARS	Myopathy, myoclonic epilepsy and psychomotor progression (167–171).	Brain and neuromuscular disorder (172–174).
2. GluRS	EARS/EPRS	Myopathy, respiratory failure, leukoencephalopathy, retinitis pigmentosa, exercise intolerance (175)	Multi-organ disorder (176).
3. LeuRS	LARS	Type-2 diabetes, premature ovarian failure and hearing loss in Perrault syndrome/ovary and ear (177).	Infantile liver failure syndrome type 1 (178–180).
4. MetRS	MARS	Myopathy (181)	Multi-organ disorder (ILD, liver failure, etc.). Pulmonary alveolar proteinosis (182, 183)
5. ProRS	PARS	Myopathy, encephalomyopathy (184, 185).	
6. SerRS	SARS	Encephalomyopathy, ataxia, mental deterioration, deafness, myopathy, exercise intolerance, MERRF, and MELAS (186).	Brain and neuromuscular disorder (187).
7. ArgRS	RARS		Multi-organ disorder, CMT (188–191)
8. CysRS	CARS		Multi-organ disorder (192).
9. ValRS	VARS		Multi-organ disorder, neurological disorders (193–196).

MERRF, Myoclonus Epilepsy with Ragged-red fibers; MELAS, Mitochondrial Encephalopathy, Lactic Acidosis and Stroke like episodes; CMT, Charcot-Marie-Tooths.

## Mechanisms of Pathogenicity

There are different ways on how aaRSs contribute to the disease pathogenesis as discussed in this review. One mechanism is due to the aaRSs being recognized as autoantigens, thus inducing an abnormal immune response. Other possible mechanisms in disease pathogenesis are impairments in their canonical or non-canonical functions due to mutations as described above.

In many systemic autoimmune diseases, it has been established that substrates of granzyme B cleavage, which is a serine protease involved in apoptosis, are more often autoantigenic rather than the native form of the protein (39, 197). Autoantigens are usually secreted or located extracellularly, in membranes, or in apoptotic blebs and contain specific structures or sequences such as coiled-coil motifs, granzyme B cleavage sites, or ELR (Glu-Leu-Arg) motifs (198). Importantly IleRS, HisRS, and AlaRS all go through granzyme B cleavage and release fragments, which contain epitopes recognized by autoantibodies (128). For example, the WHEP domain, released from HisRS upon granzyme B cleavage, which is 50 aa long, has a helix-turn-helix conformation that is also referred as a “coiled-coil”. This has been recognized as a major epitope for anti-Jo-1 autoantibodies (98). Similarly, granzyme B cleavage sites are found in GlyRS, AlaRS, and IleRS, which are autoantigens for anti-EJ, anti-PL12, and anti-OJ, respectively (8). Although it is still a mystery how aaRSs are released to the extracellular environment to accomplish all the non-canonical functions, it has been proposed that it might be through passive release from cells that are undergoing necrosis. At least five aaRSs, HisRS, ThrRS, GlyRs, TyrRs, and AsnR, have been reported to be secreted out of cells (8, 42–44, 98, 199) during tissue damage, angiogenesis, and in cancer. For the remaining four aaRSs that have been reported as autoantigens in ASSD, (i) AsnRS has also been detected in serum of healthy individuals and myositis patients possibly due to tissue damage, (ii) the levels of PheRS were reported to be associated with various types of cancer whereas there are no reports yet on the extracellular location of (iii) IleRS and (iv) AlaRS. Interestingly, some of the aaRSs are secreted or directly act on monocytes such as TrpRS, GlyRS, TyrRS, and LysRS during infections, which could be a link between infection and ASSD (85, 90–92, 139, 140).

In addition to autoantigenic motifs, mutations in genes encoding aaRSs that lead to impaired functions were reported in multi-organ disorders, neurological diseases, or cancer (**Table 3**). The most common aaRS-associated monogenic disorder is CMT, a genetic peripheral nerve disorder (8). Currently, seven known members of aaRSs (GlyrRS, TyrRS, AlaRS, HisRS, TrpRS, MetRS, and LysRS), have been implicated in CMT disease, representing the largest protein family in CMT etiology, although the role of aaRSs in this disorder is still unclear (115, 200).

## Conclusions

ASSD is an autoimmune condition characterized by the presence of autoantibodies targeting one of several aminoacyl t-RNA synthetases (aaRS) along with distinct clinical features. Although in the literature, reference to autoantibodies against 11 aaRSs can be found, autoantibodies against only eight aaRSs have been identified so far in ASSD patients as described in this review. We summarized clinical features of ASSD, what is known about ASSD-associated aaRSs, and also reviewed the known properties of the remaining aaRSs. It seems that even though the remaining aaRSs have not been confirmed to be autoantigens in diseases, their non-canonical functions inside and outside of the cell and impairment in their functions contribute to the pathogenesis of diseases such as cancer, multi-organ disorders, or neurological disorders. This strongly implicates that aaRSs have an essential role in the regulation of immune responses and more attention is needed to understand the underlying mechanisms of their pathogenic functions.

## Author Contributions

AG-F, AN, and BH: writing. AG-F and BH: figure and tables. BH: concept. AG-F, AN, BH, and IL: revision and proofreading. All authors contributed to the article and approved the submitted version.

## Funding

This work was supported by The Swedish Research Council (2020-01378), the Swedish Rheumatism Association, King Gustaf V 80 Year Foundation, King Gustaf V:s and Queen Victoria´s Freemason foundation, Funds at the Karolinska Institutet (KID), Stockholm Regional Council (ALF), Heart and Lung Foundation (2020-00380), Professor Nanna Svartz Foundation, and Karolinska Institutet Research Foundations (2020-02515). Nanna svartz: 2020-00380 KI Research Foundation : 2020-02515 The Swedish Research Council: 2020-01378

## Conflict of Interest

IL has received consulting fees from Corbus Pharmaceuticals, Inc. and research grants from Astra Zeneca and has been serving on the advisory board for Astra Zeneca, Bristol Myers Squibb, Corbus Pharmaceutical, EMD Serono Research & Development Institute, Argenx, Octapharma, Kezaar, Orphazyme, and Janssen and has stock shares in Roche and Novartis.

The remaining authors declare that the research was conducted in the absence of any commercial or financial relationships that could be construed as a potential conflict of interest.

## Publisher’s Note

All claims expressed in this article are solely those of the authors and do not necessarily represent those of their affiliated organizations, or those of the publisher, the editors and the reviewers. Any product that may be evaluated in this article, or claim that may be made by its manufacturer, is not guaranteed or endorsed by the publisher.
